# Dissecting the Genetic Diversity of USDA Cowpea Germplasm Collection Using Kompetitive Allele Specific PCR-Single Nucleotide Polymorphism Markers

**DOI:** 10.3390/genes15030362

**Published:** 2024-03-14

**Authors:** Jesse Potts, Vincent N. Michael, Geoffrey Meru, Xingbo Wu, Matthew W. Blair

**Affiliations:** 1 Department of Agricultural Sciences, Tennessee State University, Nashville, TN 37209, USA; jpotts3@ufl.edu; 2 Environmental Horticulture Department, Tropical Research and Education Center, University of Florida, Homestead, FL 33031, USA; 3 Horticultural Sciences Department, Tropical Research and Education Center, University of Florida, Homestead, FL 33031, USA

**Keywords:** cowpea (*Vigna unguiculata*), KASP-SNP, germplasm, genetic diversity, population structure

## Abstract

Cowpea (*Vigna unguiculata* L. Walp) is an important grain legume crop of the subtropics, particularly in West Africa, where it contributes to the livelihoods of small-scale farmers. Despite being a drought-resilient crop, cowpea production is hampered by insect pests, diseases, parasitic weeds, and various abiotic stresses. Genetic improvement can help overcome these limitations, and exploring diverse cowpea genetic resources is crucial for cowpea breeding. This study evaluated the genetic diversity of 361 cowpea accessions from the USDA core collection for the species using 102 Kompetitive Allele Specific PCR (KASP) single nucleotide polymorphism (SNP) markers. A total of 102 KASP-SNP was validated in the germplasm panel, and 72 showed polymorphism across the germplasm panel. The polymorphism information content (PIC) of all SNPs ranged from 0.1 to 0.37, with an average of 0.29, while the mean observed heterozygosity was 0.52. The population structure revealed three distinct populations that clustered into two major groups after phylogenetic analysis. Analysis of molecular variance (AMOVA) indicated greater genetic variation within populations than among populations. Although cowpea generally has a narrow genetic diversity, the accessions used in this study exhibited considerable variation across geographical regions, sub-species, and improvement status. These results indicated that the selected KASP genotyping assay can provide robust and accurate genotyping data for application in the selection and management of cowpea germplasm in breeding programs and genebanks.

## 1. Introduction

Cowpea (*V. unguiculata* L. Walp) is a short, warm-season legume crop native to Africa. It is one of the most popular grain legume crops in the subtropical regions across Africa, Asia, the Caribbean, and North/South America, with some production extending into Southern Europe [1]. The crop is an important source of dietary protein but also improves soil by fixing nitrogen, controlling weeds, and encouraging the growth of soil microbial communities [2]. The global production of cowpea spans over 100 countries, led by Nigeria, with an annual production of 6.5 million metric tons [3]. Biotic and abiotic challenges, including insect pests (notably: aphids and flower thrips), diseases (notably: bacterial spots, damping off, fusarium wilt, fungal leaf spots, macrophomina, root rot, and various viruses), as well as drought caused by erratic rainfall, affect production in many regions, decreasing yields and requiring costly integrated management measures. Diverse cowpea germplasm are key components for breeding new varieties addressing the biotic and abiotic challenges in cowpea production [4].

Currently, the International Institute of Tropical Agriculture (IITA) and the United States Department of Agriculture-Genetic Resources Information Network (USDA-GRIN) maintain over 20,000 cowpea accessions, including wild cowpea relatives, available to breeders and geneticists worldwide [5]. Studies evaluating seed morphology and color, along with genetic assessment, provide a more comprehensive understanding of cowpea diversity [6]. This is important for identifying desirable traits and supporting breeding efforts. Research highlights cowpea’s potential in vulnerable agroecosystems as a climate-smart protein source [6,7]. Exploring untapped diversity could enhance food security. Utilizing local, underexploited germplasm can broaden cowpea’s genetic base, which could lead to more robust varieties [6]. Analyzing genomes from multiple cowpea subpopulations helps to identify both core (conserved) and non-core (variable) genes potentially important for adaptation and breeding goals [8]. The diversity of global cowpea accessions has been reported by various researchers using DNA markers, including amplified fragment length polymorphism [9,10,11], random amplified polymorphic DNA [12,13,14], restriction fragment length polymorphism, simple sequence repeats [15], and inter-simple sequence repeats [16]. Lately, single nucleotide polymorphisms (SNP) have become the marker of choice to analyze the population structure in cowpea germplasm [17,18].

Single nucleotide polymorphisms (SNPs) markers are codominant markers, amenable to high-throughput applications, abundant in the genome, and easy and relatively cheap to assay, making them the marker of choice for evaluating genetic diversity, marker-assisted selection, genetic fingerprinting, and linkage map construction of the plant species [19,20]. In cowpeas, the use of a 1536 SNP genotyping assay from Illumina’s Golden Gate identified three population groups among 442 landraces and 46 wild African accessions [21]. The same SNP assay was used on a larger global collection of 768 cultivated cowpea genotypes representing 51 countries, resulting in three major population groups [22]. Meanwhile, in a study of 298 cowpea accessions representing a “mini-core” of the global collection at IITA, three groups were also found with 2276 SNPs [5].

To conduct rapid genotyping of populations and perform early selections in a plant species, a highly versatile fluorescent-based marker system is needed [23]. This innovative assay has been used in genetic mapping [24], and leads to marker-assisted selection [25]. KASP assays have been used for disease resistance [26], haplotype [27] and quality control [28] analyses in various crops. In cowpea, 17 KASP markers were used to successfully fingerprint parents and confirm the hybrid status of 1436 F_1_ plants derived from several breeding populations [29]. Similarly, 50 KASP markers detected significant genetic diversity and revealed unique fingerprints in 75 commercial cowpea cultivars [30].

The objectives of our study were (1) to evaluate the utility of a new set of 102 genome-wide polymorphic SNP markers in KASP genotyping assays and (2) to characterize the genetic diversity of 361 cowpea accessions representing *V. unguiculata* ssp. *unguiculata*, *V. unguiculata* ssp. *sesquipedalis*, and *V. unguiculata* ssp. *pubescens* and encompassing a wide geographical distribution. *V. unguiculata* ssp. *unguiculata* remains the most widely cultivated, providing valuable protein-rich grains utilized for its green pods as vegetable or dried as grains. *V. unguiculata* ssp. *sesquipedalis* offers a unique viny vegetable pod, that is indeterminate with long shelf life [31,32]. Lastly, *V. unguiculata* ssp. *pubescens*, though less commonly used, possesses untapped potential for enhancing breeding programs due to its potential resistance to pests and stresses [33]. *V. unguiculata* ssp *cylindrica* is used as a small-seeded fodder.

Core collections are functional starting points for gene discovery and tend to be multiplied as seed stocks with greater availability and viability than other germplasm accessions in genebanks, providing a renewable resource for researchers looking at cowpea diversity.

## 2. Materials and Methods

### 2.1. Plant Materials and DNA Isolation

A total of 361 cowpea accessions were used in this study (Table S1). This included 47 parental lines (*V. unguiculata* ssp. *Unguiculata*), of which are often used as breeding lines in cowpea breeding programs; 25 accessions belonging to *V. unguiculata* ssp. *sesquipedalis*, characterized as yard-long beans; and wild genotypes from *V. unguiculata* ssp. *pubescens*, and *V. unguiculata* ssp. *cylindrica*. The remaining 290 accessions constituted grain, fodder, and vegetable cowpea germplasm (Table S1). These accessions encompass a wide geographical distribution and were sourced from the United States Department of Agriculture Germplasm Resources Information Network (USDA-GRIN) at Griffin, GA, USA.

The seeds were planted in a 4-inch pot containing potting medium and were germinated in the greenhouse. Leaf tissue was collected from a one-month-old plant and frozen with liquid nitrogen. Genomic DNA was isolated using a previously described CTAB-based method [34]. The DNA quality was confirmed on a 1% agarose gel and quantified by spectrophotometry on a NanoDrop 2000 (Thermo Fisher, Waltham, MA, USA).

### 2.2. SNP Selection, Primer Design and Genotyping

A total of 102 SNP assays were developed for KASP evaluation in this genotyping study (Table S2). The SNP loci were chosen from 11 linkage groups to ensure a wide distribution across recombination units of a previously published cowpea genetic map [35]. All of the SNPs were high confidence calls in the Golden Gate Illumina chip assay of that previous study. The SNP flanking sequences from the study were used to produce fluorescent KASP assays for rapid genotyping at Tennessee State University (TSU). The sequences were obtained from a window 50 bp upstream to 50 bp downstream of each SNP position and were used for designing allele-specific primers for each KASP assay. Each primer contained a unique tail sequence at the 5’ end. KASP genotyping was performed according to the protocol provided by LGC Genomics Ltd. (Middlesex, UK).

The genotyping was conducted in a 384-well plate in a total of 5 μL of reaction volume. The KASP genotyping mix, comprising a 0.07 μL KASP primer assay and a 2.5 μL of 2X master mix enzyme, was dispensed by the liquid dispensing system Meridian^TM^ (LGC Ltd.) into each well that contained 50 ng of genomic DNA. The plate was then covered with an optically clear, plastic seal by the heat-based plate sealer called the Kube^TM^ (LGC Ltd.) and immediately taken to a high-throughput, water bath-based thermal cycler called a Hydrocycler^TM^ (LGC Ltd.) for polymerase chain reaction (PCR) steps.

The Hydrocycler has four separate chambers for denaturation, annealing, extension, and cooling, controlled by water bath. PCR cycling was performed using the following protocol: hot start at 95 °C for 15 min, followed by ten touchdown cycles (95 °C for 20 s; touchdown at 65 °C initially and decreasing by −1 °C per cycle for 25 s), followed by 30 additional cycles of 95 °C for 10 s; 57 °C for 60 s. The fluorescent signal was read by a FLUOstar Omega Microplate Reader (BMG LABTECH, Ortenberg, Germany) and SNP calls were made with the software program, KlusterCaller 4.1 from LGC Genomics.

### 2.3. Genetic Diversity and Population Structure

Genetic diversity statistics, such as major allele frequency, gene diversity, expected heterozygosity (*He*), and polymorphism information content (PIC) were estimated using PowerMarker 3.25 software [36]. An analysis of molecular variance (AMOVA) was performed to detect the genetic variance within and among species, geographical origins, and the entire cowpea germplasm using Arlequin 3.5.2.2 software [37].

The population structure was predicted using the program STRUCTURE version 2.3.3 [38] with 100,000 burnin length and 100,000 MCMC. The admixture model was used with the K value set from 2 to 10, with 10 iterations at each K value. The most likely value of K was determined with the Evanno test [39] as implemented in STRUCTURE HARVEST [40]. The output under the chosen K level was calculated using DISTRUCT and visualized by CLUMMP [41,42].

Phylogenetic relationships were analyzed by Bayesian maximum likelihood models using IQTREE2 with the model finder option (*MFP*) and 1000 bootstraps [43,44]. The best tree model was chosen based on the Bayesian Information Criterion (BIC). The final tree was visualized and annotated with ITOL [45]. 

## 3. Results

### 3.1. Validation of KASP-SNP Markers

The set of 102 KASP assays targeted previously confirmed SNP loci from a Golden Gate assay to develop robust markers. The genetic distance between each marker on each of the 11 chromosomes was approximately 10 cM. The average number of markers selected from each chromosome was nine, with the most markers (fifteen) selected from chromosome III and the least markers (seven) selected from chromosomes IV and VII. There were six types of SNP loci, with 67 being transitions (A/G and C/T) and 35 being transversions (C/A, C/G, T/A, and T/G).

Of the full set of KASP-SNP assays tested, a total of 83 markers (81.37% of the 102 developed) were successfully validated in the germplasm panel. Using strict criteria, a total of 72 markers (86.75% of the 83 validated) showed polymorphism across the germplasm panel and had less than 20% missing data. The variability found in the 361 cowpea genotypes for each polymorphic KASP-SNP marker was evaluated with four criteria: major allele frequency, gene diversity, observed heterozygosity, and polymorphism information content (PIC), as listed for each locus in Table 1. The PIC values for the polymorphic KASP markers in the germplasm panel varied between 0.10 (5295_403) and 0.37 (15288_527) with an average of 0.29.

All of the KASP assays that were polymorphic showed two alleles, in other words the SNPs were bi-allelic. Expected heterozygosity for these markers ranged from 0.12 to 0.95, with an average of 0.52. The more frequent allele (major allele) was listed as p or allele 1, while the less frequent allele (minor allele) was listed as q or allele 2. Major allele frequency varied from 0.51 to 0.94, with an average of 0.73. The mean genetic diversity value was 0.37, with the maximum and minimum being 0.50 and 0.11, respectively. High quality performance of the KASP assays was seen in the distribution of missing allele calls which separated them into four groups: 7 with no missing data (9400_502, 8276_369, 8408_1086, 7392_569, 7436_791, 4238_636 and 13252_298), 39 KASP markers with very low missing data (<5%), 16 KASP markers with low missing data (6% to 10%), 7 KASP markers with moderate amounts of missing data (11%–15%) (3701_796, 2728_121, 15183_436, 16011_113, 1202_1215, 11266_52 and 894_153). Three KASP markers had high amounts of missing data (16% to 20%) (5074_629, 17107_475 and 12793_473).

### 3.2. Genetic Diversity of V. unguiculata

AMOVA results for the three sub-species, *V. unguiculata* spp. *unguiculata, V. unguiculata* ssp. *cylindrical*, and *V. unguiculata* ssp. *sesquipedalis*, indicated a higher variation within the groups (97.78%) than among them (2.21%), as shown in Table 2.

While low genetic variation exists within cowpea subspecies, investigating their cross-compatibility offers a promising avenue to enrich the cowpea gene pool. Research demonstrates successful crosses, showcasing the potential to introduce valuable traits. For example, crossing early maturing and high-yielding *V. unguiculata* ssp. *unguiculata* (IT82E-124) with *V. unguiculata* ssp. *pubescens* facilitated the incorporation of insect resistance [46]. Additionally, studies have achieved success in crosses between *V. unguiculata* ssp. *unguiculata* (TVu 1) and *V. unguiculata* ssp. *sesquipedalis* (Tvu 223), particularly when the former is used as the female parent, paving the way for incorporating the long pods characteristic of sesquipedalis [47]. Furthermore, *V. unguiculata* ssp. *sesquipedalis* (Tvu-3652, Tvu-3656) readily hybridizes with *V. unguiculata* ssp. *unguiculata*, highlighting the potential for combining desired traits across subspecies [7]. These successful crosses exemplify the potential of exploring cross-compatibility within cowpea to expand genetic diversity and introduce valuable characteristics for enhanced cowpea varieties.

**Figure 1:**
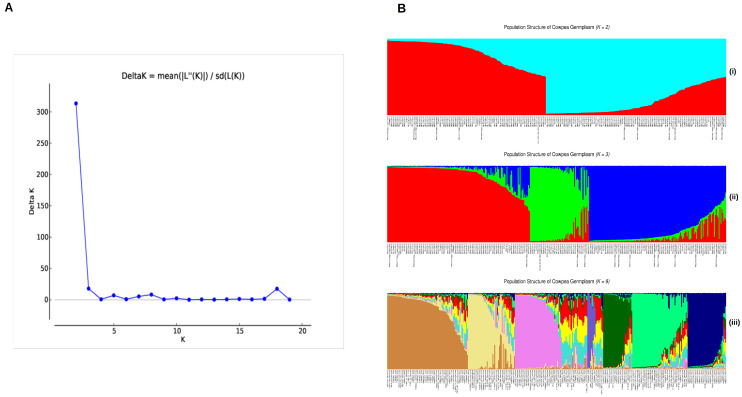
Population structure differentiation for 361 cowpea accessions analyzed with 72 KASP markers. (**A**) Plot of ΔK value with the number of subpopulations (K) from 2 to 20 based on the Evanno test. (**B**) STRUCTURE plots for K = 2, K = 3, and K = 9, with cluster assignments shown by color and each accession represented by a vertical line.

**Figure 2:**
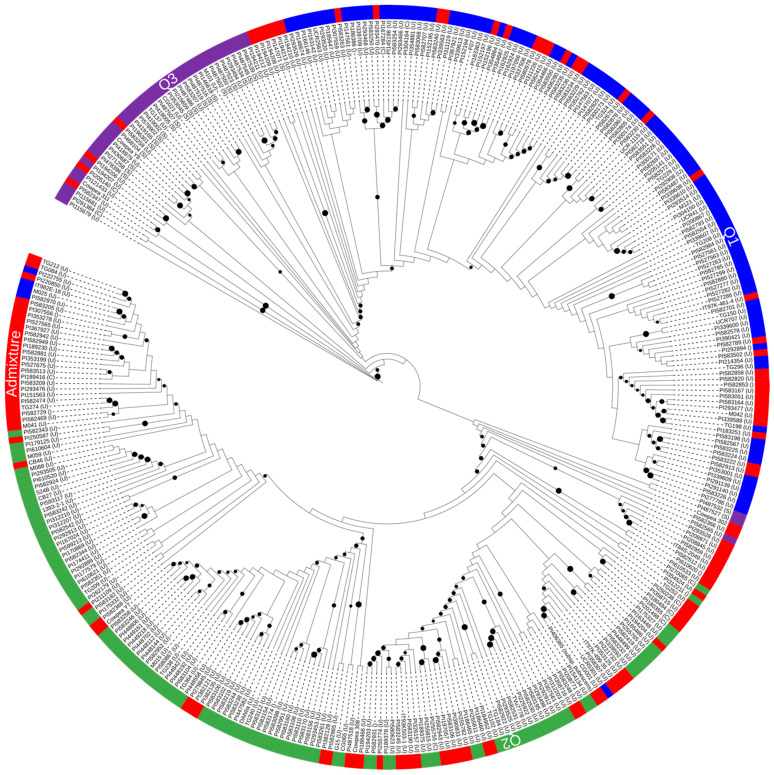
Maximum-Likelihood phylogenetic tree showing relatedness of 361 cowpea accessions.

Similar results for differences among and within groupings by regions (Europe, Latin America, Middle East, North America, East Asia, South Asia, West Asia, Southern Africa, and West/Central Africa) were observed. On the other hand, phylogenetic and population structure analyses clearly separated *V. unguiculata* ssp. *sesquipedalis* from *V. unguiculata* ssp. *unguiculata* (Figure 1 and Figure 2). However, a few accessions of *V. unguiculata* ssp. *cylindrica* were distributed within groups containing *V. unguiculata* ssp. *unguiculata* accessions.

### 3.3. Population Structure in the Global Cowpea Germplasm

The population structure of the 361 cowpea accessions was created using the most reliable set of 72 polymorphic SNPs. The Evanno test was employed to find potential subpopulations. The ΔK peak (Figure 1A) was found for K = 2 (Figure 1B(i)), which suggested a division of two major subpopulation groups. The second highest value of the ΔK graph was found at K = 3 (Figure 1B(ii))**,** which suggests a division into three groups. In both cases, the groups were partially categorized based on their taxonomy of subspecies.

At K = 2, the *V. unguiculata* ssp. *unguiculata* group and the *V. unguiculata* ssp. *sesquipedalis* group were distributed across two clusters, with the wild genotypes of *V. unguiculata* ssp. *pubescens* categorized into the first group. Despite this, the *V. unguiculata* ssp. *unguiculata* had 84 accessions in the first group (23.60% of total) and 117 in the second group (32.87% of total). The *V. unguiculata* ssp. *sesquipedalis* subspecies had six in the first group and nine in the second group. For *V. unguiculata* ssp. *cylindrica*, two were in first group and seven in second group.

At K = 3, the sub-populations differentiated the agronomic grain accessions of *V. unguiculata* ssp. *unguiculata*, the fodder accessions of *V. unguiculata* ssp. *cylindrica* and the vegetable accessions of *V. unguiculata* ssp. *sesquipedalis*. Most of the remaining accessions were identified as admixtures, indicating a genetic blend derived from the interbreeding of different subspecies or genetic sources within the accession. This is further supported by the K = 9 population structure, which differentiates genotypes based on the number of ancestral regions they derive from (Figure 1B(iii)).

Table 3 shows the distribution of genotypes by geographical origin for the division of K = 3 sub-populations. All European cowpea accessions were seen in cluster Q2, but none were found in Q1 and Q3. East Asia and Oceania accessions were only in clusters Q3 and Q1, respectively. The Middle East, East and Southern Africa accessions were in clusters Q1 and Q2 but not in Q3. The three clusters indicated that the genetic diversity and population structure of cowpea were influenced by geographic and regional factors but not individual countries of origin.

A dendrogram based on Bayesian Maximum Likelihood models was constructed and visualized with population clusters obtained through structure analysis and color coded as shown in Figure 2. The tree comprised two major clusters: the first consisted of accessions from the Middle East, Latin America, West Asia, North America, East, and Southern Africa, along with West and Central Africa; the second cluster consisted of accessions from Europe, East Asia, and Oceania. This clustering pattern underscores the significance of regional sources in shaping the genetic diversity observed in the studied populations. It is important to note that the identified clusters reflect distinct genetic relationships influenced by the geographic origin of the accessions.

Furthermore, the two clusters of the circular phylogenetic trees appeared to be generally distinct from one another: the first had Q3 (purple) and Q1 (blue), and the second contained Q2 (green) and admixtures in both (red). The *V. unguiculata* ssp. *sesquipedalis* accessions formed a sub-cluster in Q1, whereas *V. unguiculata* spp. *unguiculata* and *V. unguiculata* ssp. *cylindrical* were evenly distributed within the two major clusters, and in the admixture groups.

**Figure 3:**
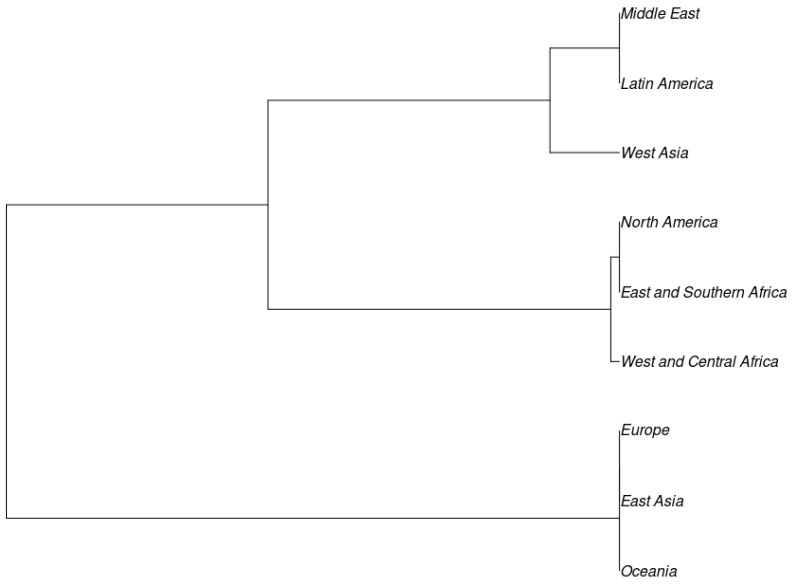
Dendrogram showing relatedness of cowpea among nine different global regions and US breeding lines based on the genetic-distance matrix using the neighbor-joining method by PowerMarker V3.25 and visualized using the software MEGA 6 (v. 3.25).

The dendrogram is consistent with clusters in the population structure study, which showed the existence of two sizable populations. The clustering did not appear to follow any pattern based on the country of origin. This was evident as shown in the dendrogram of regional sources in Figure 3.

## 4. Discussion

In this study, the effectiveness of KASP-SNP markers was validated in a cowpea germplasm panel. The results were highly encouraging, with a success rate of 81.37% (83 out of 102) markers being successfully amplified. Moreover, of the validated markers, 72 (86.75%) had high polymorphism in the panel, with less than 5% missing data. Some with 10, 15, and 20% missing data were still useful for the analysis. The success rate was similar to previous studies with Golden Gate arrays [35], where 1375 out of 1536 SNP loci (89.55%) worked in the RIL populations resulting from six crosses, testing both synthetic heterozygotes and parental genotypes for amplification and polymorphism. Another study by the same group found that 1080 SNP markers were robust across a panel of 383 genotypes [48]. Among these markers, 1054 had call rates ≥99%, while only 14 SNPs had missing data between 5 and 10%.

Despite the smaller number of SNPs utilized in the present study, the success rate was relatively similar to the previous studies [35,48]. These findings, along with the high success rate achieved in the current study, highlight the potential of utilizing a small set of effective markers in evaluating the diversity of cowpea germplasm. Consequently, researchers can confidently employ the validated KASPs in this study for genetic diversity or mapping studies, being assured of their capacity to provide dependable results across many types of cowpeas, whether these are grain, fodder, or vegetable types.

Gene diversity (He) and polymorphic information content (PIC) are both key features of a molecular marker’s ability to detect genetic variation in a population. In the current study, He (0.37) and PIC (0.29) were higher than similar studies on cowpea. Studies have reported He of 0.32 and PIC of 0.26 from 1048 SNPs evaluated on 768 accessions [22], while others observed He of 0.30 and PIC of 0.23 with 2276 SNPs evaluated on a cowpea mini-core collection (*n* = 298) maintained at IITA [5]. This indicates that the KASP-SNP markers reported here, although few, provide sufficient resources for cowpea germplasm characterization. In addition, the He and PIC values reported here indicate a substantial level of diversity within the accessions in this core collection. This is a common observation in mini-core collections of cowpea and other legumes including common bean and mung bean [49]. In this study, the Ho values were greater than He; this might be due to the composition of this core collection which was selected based on geographic origins that may lead to a defined population substructure. These findings indicate that the validated KASP-SNP markers exhibit a moderate to high level of polymorphism and were just as successful as many markers in capturing the genetic diversity within the cowpea germplasm used in this study. The marker diversity parameters show that this SNP set will be useful for future studies involving genetic diversity studies or marker-assisted breeding in cowpea.

The population structure analysis resulted in two major groups based on peak delta K = 2. The clustering observed herein did not correspond to a particular geographical origin of the cowpea accessions. Rather, the two broad groups align with two gene pools observed in domesticated cowpea [21]. A genotyping by sequencing (GBS) analysis on the same cowpea core collection with 11,283 SNP markers also revealed two groups (K = 2) categorized on taxonomy and growth habit [32], while a similar grouping (K = 2) was observed with a separate set of USDA-GRIN cowpea accessions [50]. Phylogenetic analysis indicated two broad clusters, with *V. unguiculata* ssp. *sesquipedalis* forming a group next to the wild cowpea relatives (Figure 1). All of the breeding materials from the University of California Riverside (UC Riverside) and the University of California Davis (UC-Davis) were uniformly distributed across the two clusters, but none in the *V. unguiculata* ssp. *sesquipedalis* group. This may suggest vegetable bean accessions (*V. unguiculata* ssp. *sesquipedalis*) contain unique alleles because they clustered separately upon further differentiation at K = 3 (Figure 1). Indeed, while there is a prominent level of genetic conservation amongst cowpea subspecies, some chromosomes in *V. unguiculata* ssp. *sesquipedalis* exhibit structural variations (inversion/translocation) and additional crossovers between loci [51]. However, despite this genetic distinction, successful interspecific crosses between *V. unguiculata* ssp. *unguiculata* and *V. unguiculata* ssp. *sesquipedalis* have been demonstrated [47]. This highlights the potential for exploiting cross-compatibility to introduce valuable traits, such as the long pods characteristic of *V. unguiculata* ssp. *sesquipedalis*, into cultivated cowpea varieties [7,46]. These results shed light on the genetic diversity and differentiation of cowpea germplasm subpopulations, which can be valuable for germplasm management, conservation, and breeding methods.

Analysis of genetic variation across the regions indicates that there is more diversity within each region (98.12%) than across the regions (1.87%). Similarly, most of the genetic variety exists within the species *V. unguiculata*, with a low genetic distinction between the sub-species. This is expected, and it indicates a pattern of genetic differentiation specific to geographical localities or countries due to adaptability, breeding targets, and varied utilization of cowpea in each region. Additionally, some regions have more similar cowpea accessions than others. A clustering of the cowpea germplasm according to regions in this study indicates two major clusters: the first includes Europe, East Asia, Oceania, and UC-Davis breeding lines, while the second includes the Middle East, Latin America, West and Central Africa, East and Southern Africa, West Asia, North America, and UC-Riverside breeding lines. These results indicate the presence of a significant diversity among the analyzed cowpea germplasms. While the breeding lines from UC-Riverside and UC-Davis appear to be distributed within the rest of the germplasm, *V. unguiculata* ssp. *sesquipedalis* seem to harbor different alleles in most of the SNPs analyzed. This presents a great opportunity to discover novel alleles and genes from *V. unguiculata* ssp. *sesquipedalis* for the introduction to cultivated cowpea cultivars.

Cowpea germplasm originating in different regions was found to exhibit abundant gene flow [52]. While cowpea originated in West and Central Africa, migratory routes confirm its introduction in various other regions around the world, beginning in Eastern and Southern Africa, followed by Europe and South Asia [52]. Our results indicate a progressive expansion of diversity upon the introduction of cowpeas into each region. This genetic differentiation would likely be due to the adaptation or selection, which enhanced the specific genetic diversity in each distribution area. Given the diversity of the subspecies, it is crucial that germplasm conservation efforts focus on capturing the unique diversity of each country or geographic region.

In conclusion, this study demonstrates the utility of KASP markers in analyzing the cowpea genome. In comparison to a larger collection of markers, a smaller set of properly spaced KASP-SNP markers can be cost effective and efficient in evaluating the genetic diversity of cowpea germplasm. The ability to effectively genotype and characterize germplasm collections without the requirement for first class laboratory techniques and intensive computing efforts required when utilizing large, multi-thousand marker sets have practical implications for cowpea breeding and conservation initiatives. Additionally, we suggest that a concentrated and systematic approach to marker selection can provide significant results, which might be helpful in future cowpea research and make the best use of time and resources in breeding initiatives. Future work can be conducted using the KASP markers for genetic map construction, association mapping, or marker-assisted selection in cowpea breeding programs.

## Figures and Tables

**Table 1:** Summary of 72 polymorphic KASP-SNP markers used in this study.

SNP_ID	p	q	MAF	He	Ho	GD	PIC	Missing	*X* ^2^	pval
9815_2051	0.3	0.7	0.3	0.42	0.57	0.42	0.33	0.01	46.34	0.00
9400_502	0.41	0.59	0.41	0.48	0.76	0.48	0.37	0	114.58	0.00
9263_376	0.17	0.83	0.17	0.29	0.32	0.29	0.25	0.01	4.85	0.03
8677_1492	0.1	0.9	0.1	0.17	0.17	0.17	0.16	0.02	0.14	0.71
8645_1960	0.31	0.69	0.31	0.43	0.58	0.43	0.34	0.01	47.58	0.00
8276_369	0.44	0.56	0.44	0.49	0.83	0.49	0.37	0	172.67	0.00
8408_1086	0.42	0.58	0.42	0.49	0.8	0.49	0.37	0	150.60	0.00
7953_664	0.29	0.71	0.29	0.41	0.55	0.41	0.33	0.1	38.37	0.00
8044_1006	0.27	0.73	0.27	0.39	0.49	0.39	0.32	0.01	23.99	0.00
8118_1675	0.79	0.21	0.21	0.34	0.46	0.34	0.28	0.02	32.84	0.00
8119_299	0.69	0.31	0.31	0.43	0.65	0.43	0.34	0.01	89.75	0.00
7383_1042	0.38	0.62	0.38	0.47	0.7	0.47	0.36	0.01	89.01	0.00
7392_569	0.38	0.62	0.38	0.47	0.7	0.47	0.36	0	90.41	0.00
7436_791	0.43	0.57	0.43	0.49	0.8	0.49	0.37	0	151.83	0.00
7627_617	0.43	0.57	0.43	0.49	0.81	0.49	0.37	0.01	151.77	0.00
7281_555	0.41	0.59	0.41	0.48	0.78	0.48	0.37	0.03	127.76	0.00
7034_542	0.08	0.92	0.08	0.15	0.16	0.15	0.14	0.01	0.15	0.70
7087_1100	0.15	0.85	0.15	0.26	0.27	0.26	0.22	0.01	1.83	0.18
5993_278	0.09	0.91	0.09	0.16	0.18	0.16	0.15	0.01	3.49	0.06
6796_936	0.08	0.92	0.08	0.15	0.16	0.15	0.14	0.01	2.83	0.09
699_429	0.92	0.08	0.08	0.14	0.15	0.14	0.13	0.05	0.67	0.41
673_473	0.57	0.43	0.43	0.49	0.84	0.49	0.37	0.03	183.69	0.00
5295_403	0.06	0.94	0.06	0.11	0.12	0.11	0.1	0.09	1.28	0.26
4238_636	0.43	0.57	0.43	0.49	0.82	0.49	0.37	0	165.95	0.00
4533_841	0.14	0.86	0.14	0.25	0.28	0.25	0.22	0.07	7.18	0.01
4558_472	0.24	0.76	0.24	0.37	0.47	0.37	0.3	0.04	27.25	0.00
5026_672	0.15	0.85	0.15	0.26	0.3	0.26	0.23	0.03	6.91	0.01
5074_629	0.36	0.64	0.36	0.46	0.68	0.46	0.36	0.18	69.84	0.00
5268_412	0.07	0.93	0.07	0.14	0.13	0.14	0.13	0.01	0.00	0.96
311_1536	0.11	0.89	0.11	0.19	0.2	0.19	0.17	0.09	1.02	0.31
313_221	0.08	0.92	0.08	0.16	0.16	0.16	0.14	0.02	0.16	0.69
3571_469	0.13	0.87	0.13	0.22	0.25	0.22	0.2	0.02	3.39	0.07
3701_796	0.15	0.85	0.15	0.25	0.28	0.25	0.22	0.12	3.13	0.08
3900_562	0.33	0.67	0.33	0.44	0.63	0.44	0.34	0.01	67.56	0.00
3939_496	0.16	0.84	0.16	0.26	0.3	0.26	0.23	0.04	5.25	0.02
4131_472	0.62	0.38	0.38	0.47	0.73	0.47	0.36	0.08	107.52	0.00
17107_475	0.28	0.72	0.28	0.41	0.54	0.41	0.32	0.18	33.06	0.00
2680_1095	0.2	0.8	0.2	0.32	0.39	0.32	0.27	0.07	13.53	0.00
2682_1169	0.21	0.79	0.21	0.33	0.41	0.33	0.27	0.05	20.91	0.00
2728_121	0.27	0.73	0.27	0.4	0.53	0.4	0.32	0.15	33.58	0.00
2829_305	0.25	0.75	0.25	0.38	0.48	0.38	0.31	0.09	24.62	0.00
14619_471	0.36	0.64	0.36	0.46	0.69	0.46	0.35	0.09	79.71	0.00
15183_436	0.24	0.76	0.24	0.36	0.47	0.36	0.3	0.14	24.62	0.00
15288_527	0.49	0.51	0.49	0.5	0.95	0.5	0.37	0.09	269.96	0.00
15534_890	0.28	0.72	0.28	0.4	0.54	0.4	0.32	0.07	37.32	0.00
15779_1173	0.34	0.66	0.34	0.45	0.66	0.45	0.35	0.04	77.40	0.00
16011_113	0.37	0.63	0.37	0.47	0.71	0.47	0.36	0.13	90.72	0.00
16043_314	0.23	0.77	0.23	0.36	0.45	0.36	0.29	0.05	22.39	0.00
13842_975	0.19	0.81	0.19	0.31	0.38	0.31	0.26	0.03	14.88	0.00
139_439	0.43	0.57	0.43	0.49	0.83	0.49	0.37	0.05	172.65	0.00
13987_174	0.08	0.92	0.08	0.15	0.16	0.15	0.14	0.05	0.11	0.74
14110_2536	0.38	0.62	0.38	0.47	0.72	0.47	0.36	0.05	102.41	0.00
1441_128	0.92	0.08	0.08	0.15	0.19	0.15	0.14	0.05	3.77	0.05
1202_1215	0.4	0.6	0.4	0.48	0.79	0.48	0.37	0.12	131.03	0.00
12041_453	0.31	0.69	0.31	0.42	0.59	0.42	0.33	0.01	54.81	0.00
1257_517	0.29	0.71	0.29	0.41	0.56	0.41	0.33	0.04	44.28	0.00
12793_473	0.3	0.7	0.3	0.42	0.59	0.42	0.33	0.17	46.41	0.00
12933_387	0.21	0.79	0.21	0.34	0.42	0.34	0.28	0.01	24.04	0.00
13034_542	0.14	0.86	0.14	0.24	0.26	0.24	0.21	0.07	2.45	0.12
13252_298	0.55	0.45	0.45	0.5	0.88	0.5	0.37	0	224.36	0.00
13786_529	0.29	0.71	0.29	0.41	0.54	0.41	0.33	0.03	36.26	0.00
11303_254	0.18	0.82	0.18	0.3	0.37	0.3	0.26	0.04	17.98	0.00
11470_272	0.35	0.65	0.35	0.45	0.68	0.45	0.35	0.06	85.18	0.00
11515_820	0.08	0.92	0.08	0.14	0.15	0.14	0.13	0.07	0.57	0.45
11585_1881	0.24	0.76	0.24	0.37	0.47	0.37	0.3	0.02	27.39	0.00
11683_874	0.14	0.86	0.14	0.25	0.28	0.25	0.22	0.05	7.37	0.01
11737_146	0.4	0.6	0.4	0.48	0.76	0.48	0.36	0.03	121.93	0.00
11783_1366	0.38	0.62	0.38	0.47	0.75	0.47	0.36	0.09	115.93	0.00
10466_465	0.4	0.6	0.4	0.48	0.76	0.48	0.36	0.09	118.25	0.00
10738_1400	0.27	0.73	0.27	0.39	0.51	0.39	0.31	0.07	31.52	0.00
11266_52	0.42	0.58	0.42	0.49	0.79	0.49	0.37	0.11	126.74	0.00
894_153	0.34	0.66	0.34	0.45	0.64	0.45	0.35	0.15	54.63	0.00

In population genetics analysis, allele 1 is denoted as p, allele 2 as q, MAF represents Major Allele Frequency, He signifies Expected Heterozygosity, Ho is observed heterozygosity; GD stands for Gene Diversity, PIC represents Polymorphic Information Content, Missing indicates the frequency of missing data, *X*^2^ corresponds to the CHI square value, and pval denotes the *p*-value.

**Table 2:** Analysis of molecular variance (AMOVA) results between region and species.

Regions				
Source of Variation	Sum of Squares	Variance Components	Percentage Variation	F_ST_: 0.01880
Among Groups	265.90	0.24	1.88	
Within Groups	8419.67	12.50	98.12	
Total	8685.57	12.74		
Sub-species				
Source of Variation	Sum of Squares	Variance Components	Percentage Variation	F_ST_: 0.02220
Among Groups	102.43	0.29	2.22	
Within Groups	8164.87	12.61	97.78	
Total	8267.30	12.90		

**Table 3:** Population structure analysis of cowpea accessions in clusters by number and proportion throughout the 11 geographic regions.

Regions	Number of Accessions in Each Cluster					Percentage of Accessions in Each Cluster				
	Q1	Q2	Q3	Admixture	Total	Q1	Q2	Q3	Admixture	Total
Europe	0	8	0	2	10	0	2.2	0	0.6	2.8
East Asia	0	0	18	4	22	0	0	5	1.1	6.1
Oceania	3	0	0	1	4	0.8	0	0	0.3	1.1
Middle East	3	5	0	2	10	0.8	1.4	0	0.6	2.8
Latin America	17	10	5	13	45	4.7	2.8	1.4	3.6	12.5
West and Central Africa	14	47	1	33	95	3.9	13	0.3	9.1	26.3
East and Southern Africa	34	5	0	20	59	9.4	1.4	0	5.5	16.3
West Asia	11	6	7	16	40	3	1.7	1.9	4.4	11.1
North America	22	23	8	23	76	6.1	6.4	2.2	6.4	21.1
Total	104	104	39	114	361	28.8	28.8	10.8	31.6	100

## Data Availability

Data Available upon request from corresponding author.

## Supplementary Materials

The following supporting information can be downloaded at: https://www.mdpi.com/article/10.3390/genes15030362/s1, Table S1: A total of 361 cowpea accessions were used in this study; Table S2: A total of 102 SNP assays were developed for KASP evaluation in this genotyping study.
